# Malingering

**Published:** 1888-08-04

**Authors:** W. Curran

**Affiliations:** A.M.D.


### Malingering.

By W. Curran, A.M.D.

It is not by any means common that cases of malingering occur in private practice, if we leave out of consideration those of hysterical assumption of disease. The hysterical malingerer is generally a clumsy actress, and easy to be convicted I remember being called into the front surgery of the hospital, by an excited hall porter, to see a case of hydrophobia. The patient, a young woman, who had been bitten that afternoon, was, according to her lights, indulging in an attack of furious rabies, the chief point being to bite at everything and everybody that came within her reach, to the horror of a crowd of sympathising friends. I remarked to a student that a prominent symptom in hydrophobia was wanting in this case, namely, that patients usually barked like a dog, whereupon she set up a dismal howling that "brought down the house.' Five minutes afterwards she was well. Again, I had recently under my care a young lady, aged eighteen, who manifested hysterical symptoms. I was called for several days in succession to see the state of profound coma in which she would lie for hours. On various occasions I applied the wet towel with great freedom, a galvanic battery to its full strength, and cold douches, only to find that she returned sooner or later to the same state. One morning I was summoned to her as usual, and found her in bed, apparently unconscious, and, instead of attempting to rouse her, I said to the attendant, "Never mind, let her alone," and walked out of the room. She immediately sat up and said, "Is he gone?" "Yes." "Isn't he coming back?" "No." "Then I'll get up." She had no more attacks. I once reduced a patient from strong hysterical convulsions to a very every-day state of indignation by saying, "If you knew what a fool you look you would stop that nonsense." I stayed the fit, but lost the patient.

Passing on to true malingerers, and still keeping for a few moments to civil practice, it is of course of the first importance to discover whether the patient has any object to be gained by assuming an illness or injury—whether there is a railway company to be rooked, or an accident insurance office to be swindled, or the funds of a sick club to be drawn upon, or damages for violence or negligence to be recovered. Some of these cases are of great difficulty, for there is usually some real injury on which to make a claim for compensation. The deciding on whether this injury is being exaggerated or not, whether it is the cause of the present symptoms, whether those symptoms are genuine or feigned, or how much of the suffering is real, how much imaginary, and how much counterfeit, is often a most arduous task. There can be no doubt that, in spite of any precaution that is taken, large sums of money yearly pass into the pockets of malingerers. I once had under my care a patient with an ulcerated leg, which kept its owner for three parts of a year at home at ease. Nothing would heal the wound until the time when full sick pay from his club was about to cease. Then it got well, only to break out afresh after a decent interval.

But occasionally the object is far to seek. Thus a friend of mine was recently asked to prescribe for a servant with an alveolar abscess. The face was enormously swollen, and any attempt to open the mouth caused such agony that he desisted and recommended hot fomentations. No effect being produced by this line of treatment, he, on the next occasion, succeeded in forcing open the mouth, and produced therefrom a piece of dishcloth. Here there was no object to be gained, except a temporary holiday and the sympathy of a kind-hearted mistress.

A friend, who is surgeon to a large provincial hospital, consulting surgeon to more than one railway, and who is looked on as an authority in his district in cases of injury, happens to be also surgeon to a police division. A constable fell through a trap door while on duty and injured his back. He was attended for a considerable time, and although all evidence of contusion passed rapidly away he still maintained his inability to return to duty. On the surgeon's visit he could scarcely move, but when his back was turned it was known that he got up and walked about, going out in an evening to visit his friends, etc. After some time he applied for a pension on the ground that he was incapacitated. The surgeon refused to certify, and had him carefully watched. Finding him capable of considerable exertion when his own pleasure depended on it, he at last reported him for malingering. He was then met by a certificate from a private practitioner to the effect that the man was suffering from Bright's disease, due to the injury. He had omitted to examine the urine, which contained a trace of albumen. The matter was referred to a consulting surgeon, who declined to give a definite opinion as to the origin of the disease. The man was given the benefit of the doubt, and was allowed to retire from the force with a substantial compensation, with which he immediately set up a small farm, and when last heard of was working hard in his own interests. There can be little doubt that this man was a malingerer, and that the Bright's disease was an accidental feature, perhaps of long standing.

In the army and navy, and still more in the local prisons and convict stations, the case is very different. Here escape from some irksome duty, or retirement on a pension, or a free pardon, is a goal for which a malingerer will aim with a perseverance and a stoical indifference to suffering which would, if displayed in a right direction, earn for him applause and respect. But what strikes one most forcibly is the amount of acute agony to which many malingerers will put themselves with a totally inadequate object. We can understand a soldier, whose fire of martial ardour is reduced to a mere spark, chopping off his trigger finger to avoid going into action, but we should doubt his sanity if he performed the operation to escape a single parade. Yet I have heard of prisoners permanently mutilating themselves to gain a respite from irksome employment. In the production of wounds some prisoners are really artists. Dr. Quinton relates a case of an abscess over the knee-joint which occured in a prisoner under his care; it was opened in due course, and in it was found a piece of thread which had been forced underneath the skin and the ends carefully cut off. This was done to vary the monotony of prison life by passing a few days in hospital.

A boy applied to me one morning to have his work changed, on account of an ulcer on the outside of the leg below the knee. He worked on a loom, and the position of the wound was such as to make continuance of work impossible. The ulcer was an inch and a-half long, about a quarter of an inch broad, and had obviously been made by passing some foreign body, probably a nail under the skin, and allowing it to slough out. I privately suggested to the governer that oakum picking would be the most congenial employment for the youth, and the next day the cause of the wound was made clear: he imagined that if he was taken off the loom, which he hated, he would be put to mat-making, which he liked.

There are classical methods of producing sores, which are very popular with prisoners. Among others may be mentioned the rubbing in of salt and urine, and the bandaging of a coin or piece of metal tightly over the shin-bone—the latter an easily detected fraud which no soldier, sailor, or prisoner, who respected himself, would try on any but a new hand. Mr. Smalley has given one note of a case of a soldier sent to penal servitude for twenty years who again and again manufactured abscesses by means of a worsted thread from his stocking passed deeply under the skin, and which on one occasion set up a severe attack of pyemia, and eventually albuminuria. This suffering was all undergone for such objects as to get removed from the care of some particular officer, or to be released for a time from dietary punishment. We can imagine that the process of inserting the thread beneath the skin with no better needle than a splinter of wood must have been excruciating.

*(To be continued.)*

